# Quasispecies Made Simple

**DOI:** 10.1371/journal.pcbi.0010061

**Published:** 2005-11-25

**Authors:** J. J Bull, Lauren Ancel Meyers, Michael Lachmann

## Abstract

Quasispecies are clouds of genotypes that appear in a population at mutation–selection balance. This concept has recently attracted the attention of virologists, because many RNA viruses appear to generate high levels of genetic variation that may enhance the evolution of drug resistance and immune escape. The literature on these important evolutionary processes is, however, quite challenging. Here we use simple models to link mutation–selection balance theory to the most novel property of quasispecies: the error threshold—a mutation rate below which populations equilibrate in a traditional mutation–selection balance and above which the population experiences an error catastrophe, that is, the loss of the favored genotype through frequent deleterious mutations. These models show that a single fitness landscape may contain multiple, hierarchically organized error thresholds and that an error threshold is affected by the extent of back mutation and redundancy in the genotype-to-phenotype map. Importantly, an error threshold is distinct from an extinction threshold, which is the complete loss of the population through lethal mutations. Based on this framework, we argue that the lethal mutagenesis of a viral infection by mutation-inducing drugs is not a true error catastophe, but is an extinction catastrophe.

## Introduction

The concept of a mutation–selection balance is one of the oldest and most fundamental pillars of population genetics: natural selection increases the frequency of fit variants while mutations introduce unfit variants, giving rise to an equilibrium distribution balanced between these two effects. Mutation–selection balance has been invoked to explain the persistence of undesirable genes, for example, those underlying inbreeding depression, genetic diseases, and even senescence. Despite the long history of the concept, some of its consequences were only realized in 1971, when Manfred Eigen studied mutation–selection dynamics in long genomes [1]. He found that populations do not necessarily attain classic mutation–selection balances in which the wild-type allele is most common, but rather attain an equilibrium with an abundant assemblage of mutant genotypes and a rare wild-type. He and Peter Schuster later called this collection of genotypes at equilibrium a quasispecies [2]. This concept offered not only an intuitive extension of the mutation–selection theory based on simple one- or two-locus systems, but also a novel insight into the impact of mutation rate on evolutionary dynamics. In particular, Eigen found that there are states in which a trivial boost in the mutation rate can lead to a fundamental change in the composition of genotypes in the population. This change, a phase transition in physics terms, is called the error catastrophe.

The error catastrophe has been applied liberally as a metaphor for complications of high mutation rates, as likely plagued primordial life [1] and currently challenges extant viruses with RNA genomes [3]. The error-catastrophe model inspired treatments to extirpate viral populations by mutation enhancement [4,5], and the model has been generalized to explain the attraction of populations to mutationally robust regions of fitness landscapes [6]. The error catastrophe has imparted a mystique to the quasispecies concept, and much of the literature on RNA virus evolution now uses quasispecies as an enriched synonym for a high mutation rate. An excellent and short review of the topic and its relationship to population genetics theory is provided by Wilke [7].

Eigen's insights were developed in the context of genomes with many loci, each of which suffered mutation. Appropriately, the quasispecies has since been considered in this large-genome context. Yet many of its concepts are easily illustrated in the much simpler case of few genotypes, which is our approach here. Our results are not new, per se, but our models should convey quasispecies and error-catastrophe concepts to a broad audience and correct some common misunderstandings.

## The Simplest Quasispecies

**Figure 1:**
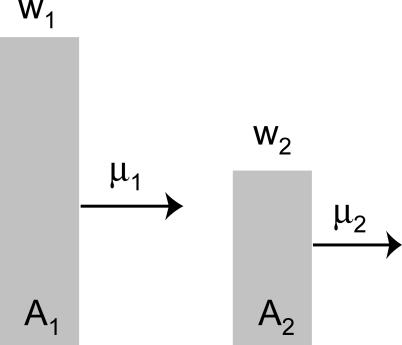
Model of Two Genotypes with Forward Mutation Each genotype *A_i_* has its own fitness *w_i_* and mutational loss μ_i_. Mutation is asymmetric, so that *A*_1_ gives rise to *A*_2_, but not vice versa.

Our basic model has the fewest number of genotypes needed to demonstrate a quasispecies and an error threshold: two [8]. Genotype *A*_1_ has fitness *w*_1_, and of those *w*_1_ offspring a fraction 1 − μ_1_ retain the *A*_1_ genotype (Figure 1). Its mutants are converted into the other genotype, *A*_2_, which has the lower fitness *w*_2_. *A*_2_ reproduces its genotype with fidelity 1 − μ_2_, and all of its mutants die.

Quasispecies concepts address equilibia, that is, the final distributions of genotypes in populations that have evolved to a stable state. In the quasispecies model, mutation and natural selection steer the population toward the equilibrium distribution, regardless of the initial distribution of genotypes. If the population does not start at the equilibrium, then mutation and natural selection steer it toward equilibrium in the quasispecies model. Mutations introduce new types with various fitnesses while natural selection causes more fit variants to increase in frequency at the expense of less fit variants. Ultimately, a population reaches a genotype distribution at which these two forces exactly cancel each other, leaving the genotype distribution unchanged. This stable assemblage of genotypes has been called both a mutation–selection balance and a quasispecies. Some authors prefer to reserve the quasispecies concept for the mutation–selection balance extreme in which the wild-type is rare [9]. This preference may stem from the property that, under a high mutation rate, the quasispecies distribution as a whole rather than a single genotype is the target of selection [2], as illustrated below.

**Figure 2:**
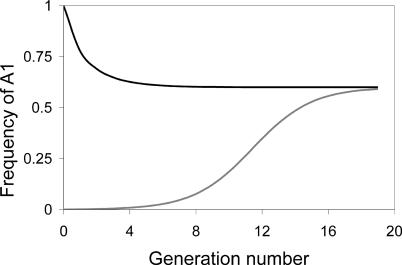
Dynamic Approach to the Quasispecies Equilibrium, for the Genotypes Illustrated in Figure 1 Drawn for *w*_1_ = 1.5, *w*_2_ = 1.0, μ_1_ = 0.7, and μ_2_ = 0.6. The upper, black curve represents a different starting condition than the lower, gray curve, but they both equilibrate to the same value.

It is essential to distinguish the process of evolution toward the mutation–selection balance from an error catastrophe, as the two are completely different. We thus begin by briefly illustrating the dynamic approach to mutation–selection balance. For any combination of mutation rates and fitnesses in our model, a population will evolve until it reaches a unique equilibrium distribution of *A*_1_ and *A*_2_. Figure 2 illustrates the evolution of two populations—one that initially consisted entirely of *A*_1_ and another that consisted almost entirely of *A*_2_. Both eventually reach the same equilibrium proportions because the mutation rates and fitnesses are the same for both cases. This end-state is the mutation–selection balance, whereby mutation continually creates *A*_2_ from *A*_1_ and natural selection continually purges *A*_2_ in favor of *A*_1_; it is also a quasispecies where *A*_1_ is the wild-type and *A*_2_ is the mutational “cloud” that persists with it. In a strict sense, equilibrium is reached only after infinitely many generations, but the deviation from equilibrium quickly becomes insignificant.

If a mutation rate or fitness is changed, the equilibrium distribution changes. Thus, the analogous figure for a different equilibrium would have the curves converging to a different value on the vertical axis. In particular, if we continually lower the fitness (*w*_1_) or the mutation-free fraction of offspring (1 − μ_1_) of *A*_1_ relative to that of *A*_2_, then the equilibrium distribution will move to progressively lower values on the vertical axis, until it lacks *A*_1_ entirely. This brings us to the error catastrophe.

## Error Catastrophe: The “Loss” of *A*_1_ at Equilibrium

Error thresholds and error catastrophes are properties of mutation–selection equilibria, and an error catastrophe is a specific type of change in the equilibria. Consider again the genotypes and fitnesses illustrated in Figure 1. The product *w*_1_(1 − μ_1_) is the number of *A*_1_ offspring of an *A*_1_ parent; we will refer to this product as the replacement rate of the genotype—the product of fitness times the mutation-free fraction of offspring. For mathematical simplicity, we assume that this number is an absolute quantity greater than one and that it does not change with population density. Hence, the population is forever expanding in unlimited space (this assumption is convenient but not necessary). For convenience, we will continue to treat *A*_1_ and *A*_2_ as single genotypes, but later in the paper, we will explain how they can be treated as sets of genotypes and how additional types can be included (e.g., *A*_3_, *A*_4_, ...).

Since *A*_1_ has a higher fitness than *A*_2_, it follows that, for small mutation rates μ_1_, the replacement rate of *A*_1_ is higher than the replacement rate of *A*_2_, or *w*_1_ (1 − μ_1_) > *w*_2_ (1 − μ_2_). As long as the replacement rate of *A*_1_ exceeds that of *A*_2_, both genotypes will be maintained at equilibrium: *A*_1_ because of its higher replacement rate, and *A*_2_ because of mutation from *A*_1_. If the mutation rate of *A*_1_ increases such that the mutation-free fraction of *A*_1_ drops relative to that of *A*_2_, however, the replacement rate inequality will eventually reverse, yielding a simple error catastrophe: *A*_1_ is no longer maintained. The error threshold is the point at which both genotypes have identical replacement rates. Beyond this point, *A*_2_ has the higher replacement rate, and *A*_1_ is absent at all equilibria—the lack of back mutations ensures that it is not recreated from *A*_2_.

**Figure 3:**
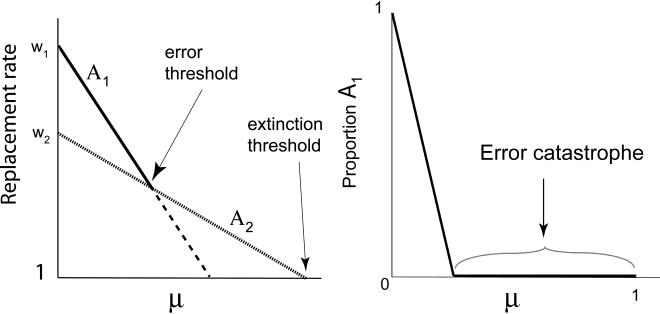
The Error Threshold and Error Catastrophe Left: replacement rates of the two genotypes in Figure 1, *w*_1_(1 − μ_1_) for *A*_1_ (solid black line extending to dashes) and *w*_2_(1 − μ_2_) for *A*_2_ (gray line). Here we let μ_1_ = *k*μ and μ_2_ = μ, with *k* > 1 so the two replacement rates can be plotted as lines in the same plane. Since *w*_1_ > *w*_2_, the constraint on *k* ensures a higher replacement rate of *A*_1_ than of *A*_2_ at low mutation rates and the reverse at higher mutation rates. The point at which the lines intersect is the error threshold, beyond which *A*_1_ is absent, hence the use of dashes for this part of its replacement rate function. If replacement rate drops below unity, the population goes extinct, so the functions are not extended below one on the vertical axis. Right: the frequency of *A*_1_ declines as the mutation rate increases until the error threshold is reached. At higher mutation rates, only *A*_2_ is present. The decline in the frequency of *A*_1_ with μ toward the error threshold may be linear (as shown here), concave, or convex, depending on parameter values. (Right side drawn for *w*_1_ = 3.0, *w*_2_ = 2, and *k* = 2.)

The nature of this simple error threshold and error catastrophe is easily visualized (Figure 3). The left part of the figure shows *A*_1_ and *A*_2_ replacement rate functions approaching each other as the mutation rate increases toward the error threshold. The functions cross at the error threshold, giving rise to an error catastrophe (complete loss of *A*_1_) for higher mutation rates. The right part of the figure illustrates that, despite the suggestive terminology (threshold and catastrophe), an error catastrophe is the culmination of a gradual decrease in the equilibrium frequency of *A*_1_ rather than a dramatic one. An apt analogy is the geological transition from mountains to plains: starting from the mountains, the plains are approached gradually until they are reached, at which point the plains continue in the absence of any trace of mountains. (Note, however, that a true phase transition does occur at the error threshold, as has been shown by Leuthausser [10,11].) The equilibrium proportion of *A*_1_ approaches zero as the mutation rate approaches the error threshold. Just before the threshold, the population is dominated by the “cloud” of *A*_2_ mutants around a tiny fraction of *A*_1_, and thus fulfills the standard criteria for a quasispecies.

An error threshold exists because deleterious mutations impact some genotypes more than others (reviewed in [7]): in this model the threshold is breached only if *A*_1_ experiences a potentially much greater mutational loss than *A*_2_. Beyond the error threshold this deleterious effect of mutation is retarded: by replacing a mutation-sensitive genotype with a mutation-robust genotype, the error catastrophe reduces the speed at which the replacement rate drops in response to increases in the mutation rate [7]. This outcome can be seen in the left side of Figure 3: the *A*_1_ replacement rate function is steeper than the *A*_2_ replacement rate function, so increases in the mutation rate have less impact after the error threshold. (Not only do the functions depict genotype replacement rate, but the uppermost functions also give the mean replacement rate in a population at equilibrium.) Below we will specifically describe how such mutational robustness may be achieved; we have depicted an error catastrophe between two genotypes, but the logic is easily extended to larger sets of mutationally connected genotypes. Again, we emphasize that an error catastrophe is not a process per se; rather it is a change in the equilibrium distribution of genotypes arising under a relatively high mutation rate. If the mutation rate of a population is increased from below to above an error threshold, however, then there will be a dynamical loss of *A*_1_ as the population approaches the new equilibrium.

Three implications of this analysis are significant. First, an error catastrophe is not equivalent to a population extinction. Second, fitness landscapes may contain multiple error thresholds. Third, the error threshold is affected by finite population size, the inclusion of back mutation from the mutant to the wild-type, and recombination. We now discuss each of these in detail.

### 

#### Error catastrophes versus population extinction.

High mutation rates can cause extinction, but not as an error catastrophe (Figure 3). An error catastrophe takes place when one genotype's replacement rate exceeds another's and thus displaces it at the equilibrium—a replacement of one self-sustaining genotype by another self-sustaining genotype. The population cannot maintain the genotype with the higher fitness, *A*_1_, since its high mutation rate causes it to have a lower replacement rate than *A*_2_. Thus, the error threshold refers to the loss of the highest fitness genotype in favor of other genotypes with lower fitness but greater mutational robustness.

In contrast, extinction occurs at the point that the best genotype cannot reproduce itself to maintain a minimum population size (*w_i_*[1 − μ*_i_*] < 1 for all genotypes). The most trivial example of the distinction between an error threshold and an extinction threshold is the case in which only one genotype exists, and all mutations are lethal. No error catastrophe is possible because no other genotypes exist, but extinction is obviously possible. Our model is one genotype more complicated: mutant offspring of *A*_1_ survive as *A*_2_, but mutants of *A*_2_ are inviable. Thus, there is not an additional error threshold beyond *A*_2_, because the model does not allow a third inferior genotype. An *A*_2_ population, however, will go extinct if its net growth rate drops below one (the horizontal axis in Figure 3). While an error threshold depends on the relative replacement rates of the genotypes, the extinction threshold depends on the absolute replacement rates of the genotypes.

**Figure 4:**
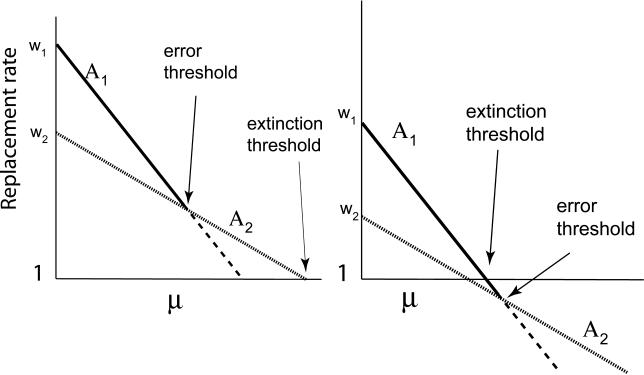
Extinction versus Error Catastrophe Left: the error threshold is crossed at a lower mutation rate (μ) than the extinction threshold. Right: the extinction threshold is crossed before the error threshold. Otherwise as in Figure 3.

There are several possible relationships between error thresholds and extinction thresholds. The genotype with highest fitness in this model (*A*_1_) can disappear through either an error catastrophe or a population extinction, but *A*_2_ can disappear only through extinction. Depending on the values of *w*_1_, *w*_2_, and μ_2_, the rate of mutation μ_1_ at which *A*_1_ would be lost to *A*_2_ in an error catastrophe may be less than or greater than the value of μ_1_ at which the population would go extinct. If the error threshold lies beyond the extinction value, then as μ_1_ increases, the *A*_1_ + *A*_2_ population will go extinct of its own accord before a true error catastrophe can happen, because the population has disappeared before the mutation rate rises high enough for the error catastrophe (Figure 4, right). If the opposite is true, then *A*_1_ will be displaced by *A*_2_ in an error catastrophe before the replacement rate of either type drops below one (Figure 4, left).

#### Multiple error thresholds.

**Figure 5:**
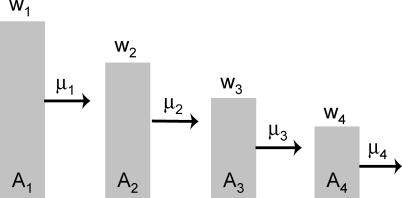
Model of Several Genotypes with Forward Mutation Each genotype *A_i_* has its own fitness *w_i_* and mutational loss μ*_i_* so that error thresholds can exist between each pair of genotypes.

The model implies that multiple error thresholds may exist in a fitness landscape. For example, a fitness landscape may include multiple *A_i_* with associated *w_i_* and μ*_i_* (Figure 5). With an appropriate hierarchy of mutation rates, error thresholds could operate sequentially, so that as mutation rates increase, the population drops from one *A_i_* to the next, then to the next, and so on [12]. It is also possible for an error catastrophe to jump over an intermediate peak [13]. For example, if the mutation rate at which the error threshold is crossed between *A*_1_ and *A*_2_ exceeds the mutation rate for the error threshold between *A*_2_ to *A*_3_, then a transition will occur from *A*_1_ to *A*_3_, skipping the intermediate in which *A*_1_ has disappeared but *A*_2_ remains (see below). The interplay between extinction catastrophes and error catastrophes can likewise add interesting complexity to the model.

#### Implications of finite population size, back mutation, and recombination.

Our model assumes deterministic dynamics and ignores the stochastic effects of finite population sizes. We have argued that populations will equilibrate either to a combination of *A*_1_ and *A*_2_ or to *A*_2_ exclusively. This equilibration strictly requires an infinite amount of time in an infinite population. In a finite population, as long as the effective population size times the mutation rate is significantly bigger than one (*N*μ ≫ 1), the finite population behaves similarly to the infinite population, except that the error threshold is not as sharp. In particular, the lack of back mutations ensures that random events will eventually cause the loss of genotype *A*_1_ from the population, so that only genotype *A*_2_ remains. If *A*_2_ represents multiple types of mutants, then this random loss of *A*_1_ is Muller's ratchet [14,15]. Beyond the error threshold, this transition is simply much faster, because selection favors it. The threshold is also altered by the introduction of back mutations from *A*_2_ to *A*_1_, as described in Box 1.

**Box 1. Back Mutation**

**Figure 6:**
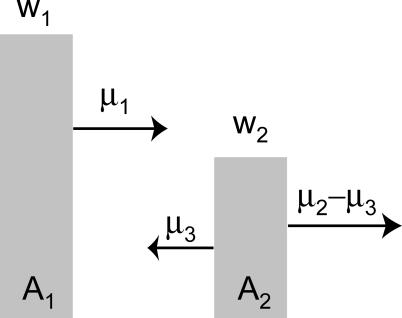
Model of Two Genotypes with Back Mutation, μ_3_ As in Figure 1, except that some of the mutations away from *A*_2_ recreate genotype *A*_1_. The total mutation rate of *A*_2_ is still μ_2_, so the back mutation rate cannot exceed this value (i.e., μ_3_ ≤ μ_2_).

Our model of two genotypes ignored back mutation, hence *A*_1_ could not be regenerated once lost from the population (see Figure 1). The absence of back mutations is often assumed in models of population genetics (e.g., the “infinite alleles” and “infinite sites” models of population genetics [42,43]). This assumption offers analytical convenience and is perhaps an approximate interpretation of asexual genomes, in which an “allele” can be considered the entire suite of mutations in a genome; a new mutation, which can occur anywhere in the genome, is unlikely to recreate a former allelic state. Nonetheless, since back mutations can occur in many systems, it is useful to study their effect on the error threshold. In particular, they present a special challenge to the concept of an error threshold: when back mutations occur, the wild-type genome is never strictly lost in a deterministic sense. What, then, is the counterpart to a strict error threshold? To approach this question, we return to the simple model in Figure 6.

At this point we use simple matrix algebra to explore the problem. Assuming discrete time steps, let *n_i_* be the number of individuals with genotype *A_i_*; mutation precedes reproduction, so the fitness *w_i_* accrues to an individual after its genotype experiences any mutation (the model is just as easily constructed with mutation occurring after reproduction). Two equations describe the system:

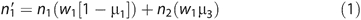


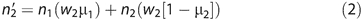
The system can then be described with the aid of the transition matrix:

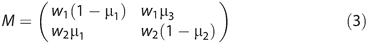

so that 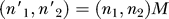 . The only new parameter with respect to our earlier model is μ_3_, which allows for back mutation and cannot exceed μ_2_. If we set μ_3_ = 0, then these equations describe our earlier model exactly (in Figure 1). To evaluate the long term behavior of the system, we consider *n*_1_(*t*) and *n*_2_(*t*) as *t* becomes arbitrarily large; that is, we calculate (*n*_1_, *n*_2_) *M^t^* for large *t*. The standard method for evaluating long-term behavior in such a linear system is to consider the eigenvalues of the system, found as the values of λ satisfying

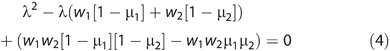

To establish a baseline, we first assume no back mutation (μ_3_ = 0). Equation 4 then yields the solutions

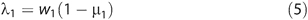

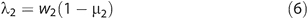

These two eigenvalues are the respective replacement rates of *A*_1_ and *A*_2_ described in Figure 3. Importantly, λ_1_ is larger for some mutation rates, but λ_2_ is larger for others, and it is the intersection of the eigenvalues that gives rise to the error threshold. This simple picture appears to change fundamentally with even a small level of back mutation. When μ_3_ > 0, the two eigenvalues become

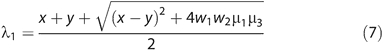

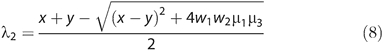

where *x* = *w*_1_(1 − μ_1_) and *y* = *w*_2_(1 − μ_2_).

**Figure 7:**
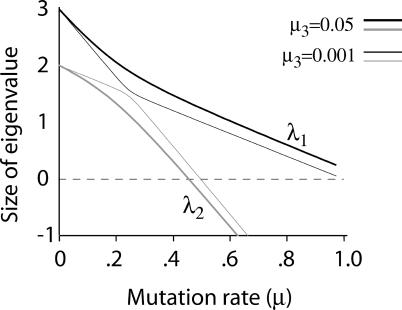
Eigenvalues When a Small Level of Back Mutation Occurs In contrast to the case of no back mutation in Figure 3, the eigenvalues here do not cross but change slope as they approach each other. The dark curves (upper) correspond to the maximum eigenvalue (λ_1_) and the light curves (lower) to the smaller eigenvalue. The thick, outer curves are drawn for a back mutation rate of μ_3_ = 0.05, and the thin, inner curves for μ_3_ = 0.001. The effect of increasing back mutation is clearly seen as increasing the minimum distance between the eigenvalue functions. Other parameters are the same as for the right side of Figure 3: *w*_1_ = 3.0, *w*_2_ = 2, and *k* = 2.

Whereas a transition from λ_1_ to λ_2_ as the dominant eigenvalue occurred at *x* = *y* in the absence of back mutation, now λ_1_ remains superior at all values of *x* and *y*. As μ_3_ approaches arbitrarily close to zero, the eigenvalues approach each other but do not cross. Thus, there is no strict error threshold unless μ_3_ equals zero exactly. Yet as μ_3_ approaches zero, the system appears more and more like that with no back mutation (Figure 7). So the mathematical discontinuity between systems with and without back mutation does not obviously translate into a meaningful biological distinction, but large amounts of back mutation do change the picture.

When *N*μ ≪ 1, the above description does not hold. Mutations occur so rarely, that the population is very likely to fix or lose each mutation before the next one arises. In this case, it no longer makes sense to talk about a quasispecies—there is no mutation–selection balance, but instead a fixed type, the wild-type, with possibly some recent mutants. The wild-type essentially undergoes a random walk in genotype space, in which it tends to “hang out” more often in areas with high fitness [16]. Sella and Hirsh [16] showed that population size rather than mutation rate is the critical randomizing factor: the smaller the population size, the less the population is able to maintain genotypes with high fitness.

We ignored the effect of recombination, as does most of the literature on the error catastrophe. The effect of recombination on the quasispecies can be very complicated. A surprising result is that recombination can make a genotype more vulnerable to an error catastrophe, causing the error threshold to occur at a lower mutation rate [17]. Although a full explanation of this effect does not seem to have been provided, one contributing factor is that, close to the error threshold, the quasispecies contains mainly mutants, and very few individuals of type *A*_1_. Therefore, most individuals of type *A*_1_ will mate with those of type *A*_2_. If our model is interpreted as a single-locus model, recombination would make no difference. But if *A*_2_ is a collection of genotypes with mutations at possibly many loci and *A*_1_ is the mutation-free class, then recombination could decrease the rate at which *A*_1_ parents have *A*_1_ offspring and thus cause the error threshold to occur earlier [17]. Recombination might also operate in the reverse direction, however, if *A*_2_ genotypes frequently recombine to create *A*_1_ offspring. A greater exploration of this problem is warranted.

## Survival of the Flattest

Error thresholds require that some genotypes or phenotypes are more sensitive to mutation than others. Biologically, many phenotypes have the property that they can be produced by multiple, mutationally related genotypes, and mutational robustness is known to vary among protein and nucleic acid sequences [13,18–22]. The set of genotypes for a particular phenotype is called its neutral network [23]. Since mutations within a neutral network preserve the phenotype, this genetic redundancy can reduce the phenotypic mutation rate without altering the underlying genetic mutation rate. As discussed above, a lower rate of phenotypic mutations (e.g., *A*_1_ to *A*_2_) leads to a higher net replacement rate of the phenotype.

Here we illustrate how the breadth of a neutral network—the number of and mutational connectivity among genotypes contained within it—affects the competitiveness of the phenotype. In particular, “survival of the flattest” refers to the ability of inferior phenotypes with large neutral networks to displace superior phenotypes with smaller neutral networks. This phenomenon was recently demonstrated in digital life simulations and simulated RNA molecules [6,24].

**Figure 8:**
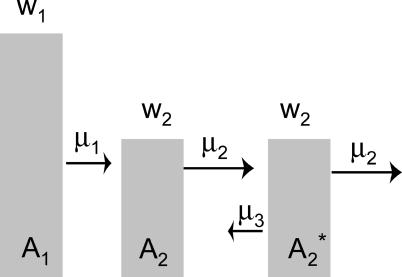
Model of Three Genotypes with a Simple Neutral Network Both *A*_2_ genotypes have the same fitness and the same forward mutation rates μ_2_. The error threshold with μ_3_ = 0 is the same as in Figure 1. As μ_3_ increases, the *A*_2_ network becomes more competitive against *A*_1_, and thus the error threshold value of μ_1_ decreases. Equations for this system can be derived and analyzed with similar methods as in Box 1.

**Figure 9:**
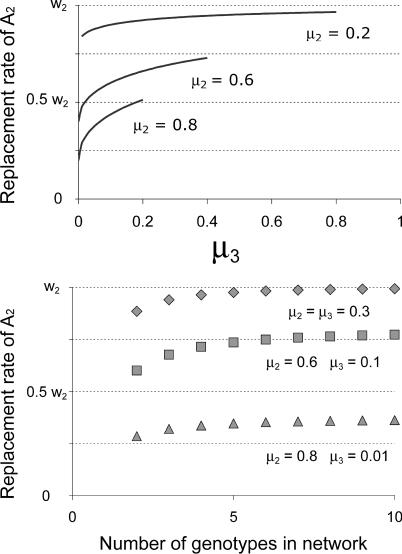
Network Size and Mutational Connectivity Affects Replacement Rate Top: effect of back mutation (μ_3_) between the pair of *A*_2_ genotypes in Figure 8. The vertical axis gives the replacement rate of the *A*_2_ network. Since all *A*_2_ genotypes are mutationally interconnected, they ultimately grow at the same rate in the limit. In the absence of back mutation, the neutral network has the replacement rate *w*_2_ (1 − μ_2_), and replacement rate increases as back mutation is increased up to its maximum of μ_3,max_ = 1 − μ_2_. (The replacement rate function is obtained as the eigenvalue associated with *A*_2_ in the transition matrix.) Bottom: the replacement rate of the neutral network also increases with the number of genotypes in it. Figure 8 shows the network with two genotypes; third and additional genotypes would be added so that each is mutationally balanced: each additional genotype loses μ_2_ of its progeny to become the genotype to its right and loses μ_3_ of its progeny to become the genotype to its left, but it receives μ_2_ mutants from the genotype to its left and receives μ_3_ mutants from the genotype to its right. The benefit of increasing the network size is shown for three different combinations of forward and back mutation rates.

We explore the impact of neutrality on the error threshold with a simple extension of our previous model, this time allowing two genotypes to have the phenotype *A*_2_ (Figure 8). This creates a minimal neutral network of two genotypes. In the absence of back mutation (μ_3_ = 0), the error threshold is unaffected by the “network” and is the same as with a single *A*_2_ genotype: *A*_2_ here has exactly the same properties as the *A*_2_ in Figure 1, so the *A*_1_/*A*_2_ error threshold is the same as in Figure 1. Yet the error threshold does change when back mutation is allowed from the second *A*_2_ genotype (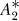 ) to the first (*A*_2_). Back mutation provides a mutational “connectivity” among both genotypes in the neutral network and renders the network more competitive against *A*_1_. This connectivity serves to bolster the frequency of the left *A*_2_ genotype at the expense of the right *A*_2_. Since only the right peak experiences mutations off the neutral network (at rate μ_2_), this shift increases the overall replacement rate of *A*_2_, allowing *A*_2_ to more easily supplant *A*_1_. Higher rates of back mutation increase this effect (Figure 9, top) and also retard the extinction of *A*_2_. (As we discuss later, allowing an infinite succession of *A*_2_ genotypes without back mutation has the same effect on the error threshold as abolishing the forward mutation rate of a single *A*_2_ genotype, since the population will never mutate off the *A*_2_ neutral network.)

This model can also be extended to demonstrate the impact of neutral network size on the error threshold. In this case, we add *A*_2_ genotypes into the middle of the neutral network, each with forward mutation rate μ_2_ and back mutation rate μ_3_. Figure 9 (bottom) shows that as the number of genotypes increases, the replacement rate of the *A*_2_ network also increases, thus shifting the error threshold in favor of *A*_2_ and buffering *A*_2_ against extinction.

In summary, the error threshold decreases as the fitness landscape around the inferior phenotype broadens, either via the extension of the neutral network to include additional genotypes or via a net decrease in mutation off the network. The error threshold above which a particular wild-type is lost will depend on its fitness relative to the fitness and breadth of the inferior network. The broader and more mutationally connected the inferior neutral network, the greater its tolerance for mutation and the more likely an error catastrophe that tips the population in its favor. Nonetheless, a sufficiently high fitness can compensate for a high rate of mutational loss.

**Figure 10:**
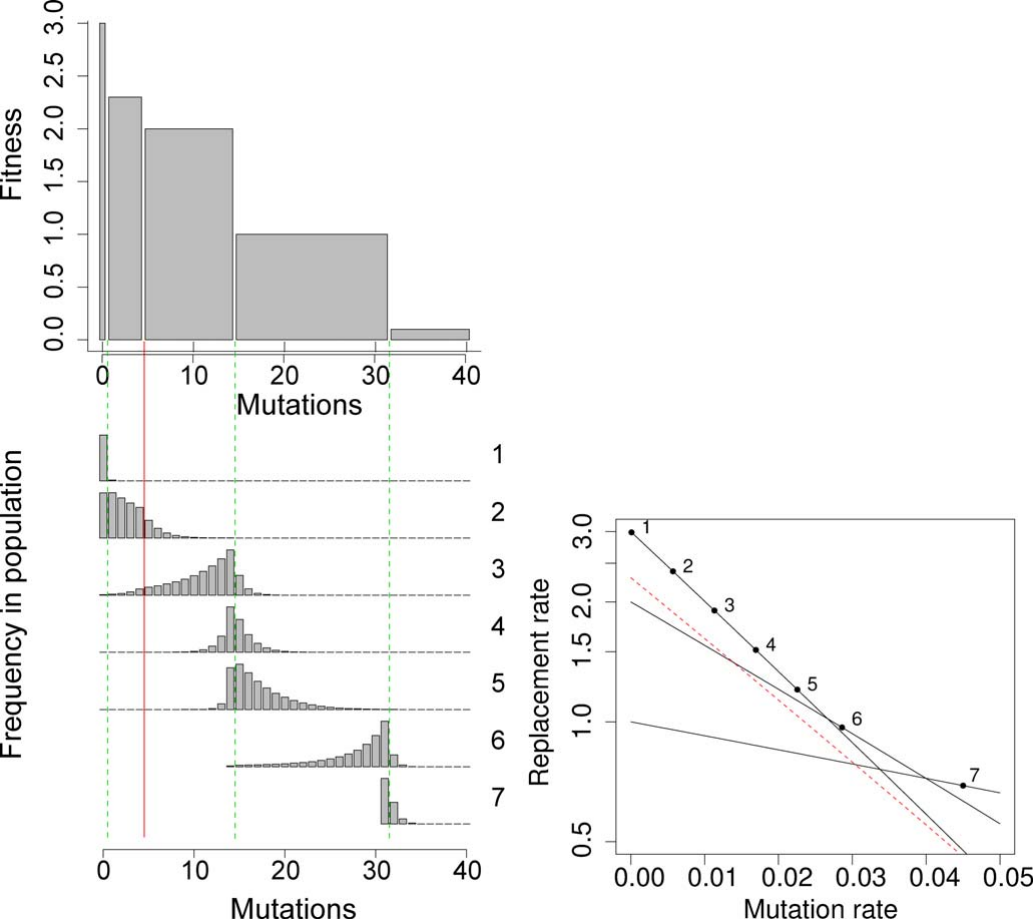
Multiple Error Thresholds on a Binary Sequence of Length 40 Each allele has two states—wild-type and mutant. The per base mutation rate from wild-type to mutant is identical for all loci. Back mutation from mutant to wild-type is not allowed. As mutant alleles fix in the population, the proportion of the sequence that can still mutate decreases. Multiple error thresholds exist because the effective mutation rate per genome drops as mutations accumulate. The model is otherwise as in Figure 1 and Box 1, and as specified below. Top: the fitness landscape. The *x*-axis shows the mutation distance from the “perfect” genotype consisting of all wild-type (zero mutant) alleles. The *y*-axis shows the fitness. Lower left: equilibrium distributions of genotypes in the population for seven mutation rates from 0.0001 to 0.045. In all plots the *x*-axis indicates the number of mutations in the genome and the *y*-axis indicates frequency in the steady-state population; the shortest bars merely indicate the presence of genotypes at some (low) frequency, and the absence of a bar indicates absence of that genotype from the equilibrium population. Plots 1–5 show equilibria before the first error catastrophe, that is, for mutation rates that are less than the first error threshold. Plot 6 shows an equilibrium after the first error catastrophe and before the second error catastrophe, and plot 7 shows an equilibrium after the second catastrophe. The vertical dashed green lines indicate the stable networks that can attract error catastrophes. These correspond to the first (wild-type), third (5–14 mutations), and fourth (15–31 mutations) networks, starting from the left. The second network, with 1–4 mutations (represented by a red line), is never stable as the fittest type in the population, and is skipped. Lower right: replacement rates of the various fitness plateaus. Numbers correspond to panels in the figure to the left. The *x*-axis shows the mutation rate; the *y*-axis shows the growth rate (with a log scale). One can see two error thresholds. The red dashed line represents the replacement rate of the second network, with 1–4 mutations, and shows why this plateau never stabilizes—it is too narrow relative to its fitness advantage.

These trade-offs also apply to more complex fitness landscapes consisting of multiple neutral networks with differing fitnesses. In this case, the sequence of error thresholds becomes difficult to intuit and has not previously been addressed quantitatively. Figure 10 illustrates a succession of error catastrophes in a single landscape with varying widths and heights of neutral networks, for a genome with 40 loci. In general, the narrower networks are skipped as the error catastrophes move to the flatter networks. In this particular model, there is no back mutation. Error thresholds exist because the genomic mutation rate is proportional to the number of unmutated loci, hence the phenotypes of lower fitness experience lower overall mutation rates.

## Comparison to Eigen's and Other Models

For those familiar with Eigen's original quasispecies model ([1], a simplified version appears on p. 480, Table 8) and more recent versions applied to nucleotides [2,8,25], we relate it to our simpler model using our notation. In those models, a genome consists of *L* loci with a per locus mutation rate of *ν*. A single mutant-free, wild-type genotype *A*_1_ has highest fitness *w*_1_, and all other genotypes with one or more mutations fall into the same neutral network in which each genotype has the same fitness *w*_2_. Eigen's classic error threshold result is that, depending on *L* and *w*_1_/*w*_2_, there is a specific per base mutation rate *ν* beyond which the wild-type is lost or maintained only through back mutation and the *w*_2_ neutral network prevails, as in our Figure 10 (the most-fit genotype is maintained if back mutation is allowed, as in Box 1).

A second class of models is more challenging to fit into the current framework [15,26]. In those models, a linear series of genotypes is connected by forward mutation but not back mutation (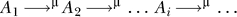 ) -->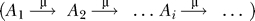 , and all genotypes have the same mutation rate. An error threshold exists in those models but not in ours. One critical difference is that those models include infinitely many genotypes. Thus, if our *A*_1_/*A*_2_ model is restricted to a single forward mutation rate for all genotypes, an error threshold appears only when infinitely many *A*_2_ genotypes exist. In this extreme, the *A*_2_ network no longer experiences any mutational loss, and hence its mutation rate effectively disappears. The existence of an error threshold in those models also requires that the fitnesses of all of the genotypes be greater than some positive value [15]. Thus, those models also appear to be consistent with our framework.

## RNA Viruses, Quasispecies, and Lethal Mutagenesis

The error catastrophe has found its greatest appeal in work on RNA viruses, whose genomes have high mutation rates. One appeal of the quasispecies concept in this work is that it can potentially explain the high levels of sequence variation observed in RNA virus samples: the high mutation rate of RNA genomes should lead to a mutation–selection balance with lots of variation. Yet despite the frequent reference to quasispecies concepts in the literature, relatively few studies have demonstrated that observed variation is truly a quasispecies equilibrium [9]. An early study of the RNA phage Qβ reported that sequence variation in a population was high but approximately stable over time around a consensus sequence, supporting the basic concept of a quasispecies [27]. There are now countless studies revealing population variation in RNA viruses of eukaryotes, but one cannot easily determine whether the variation stems primarily from mutation–selection balance or from the simple alternative of selection for different genotypes within the spatially heterogeneous environment of the host [9]. Part of the difficulty is that quasispecies models consider mutation only as a degenerative process reducing replacement rate. They do not address mutation as an exploratory process for finding new fitness peaks—a process that must obviously be considered when interpreting empirical data. Quasispecies models also tend to ignore population structure and migration.

Further support for quasispecies behavior was provided by an empirical study of the dynamical approach toward quasispecies equilibrium. Burch and Chao [28] obtained a high-fitness isolate of the RNA phage φ6. Upon propagation in the same environmental conditions, however, the fitness of the viral lineage declined to the level of culture from which the isolate was originally obtained. Their interpretation, with which we agree, was that the initial isolate was a somewhat rare, high-fitness type from a quasispecies distribution, and that it re-evolved the equilibrium quasispecies distribution on further passage. It thus seems clear that some RNA virus populations satisfy the basic tenets of quasispecies theory, even if based on relatively few studies.

Quasispecies error thresholds have been invoked in an important medical application, the extinction of RNA virus populations by lethal mutagenesis—elevating mutation rates with base analog drugs to the point that the virus population disappears. In 2005, an entire issue of *Virus Research* was devoted to this topic (volume 107, issue 2). Base analogs are incorporated into the viral genome as it is being copied from the parent strand. In general, incorporation of a base analog can have two types of effects: chain termination, resulting in immediate death of the progeny strand, or mutation, whereby the base analog is later miscopied when the genome gets replicated. The latter mechanism underlies the antiviral activity of the drug ribavirin for some RNA viruses [4,29,30]. It is commonly argued that the extinction of the viral population by mutagenesis is an example of an error catastrophe (e.g., [4,5,30–32]). Others have argued against this interpretation [7], and our models illustrate clearly that extinction is distinct from an error catastrophe, and hence that lethal mutagenesis does not necessarily produce an error catastrophe.

From a practical perspective, it does not matter whether lethal mutagenesis is referred to as an error catastrophe or extinction catastrophe. There may be a benefit, however, to distinguishing between these two concepts and, more generally, to clarifying the concept of a quasispecies. For example, consider a protocol of sublethal mutagenesis, whereby a virus mutation rate is artificially elevated close to an extinction threshold but the virus population persists. Conventional wisdom suggests that such a strategy should be disastrous from a host health perspective, because the increased mutation rate may accelerate viral adaptation. Yet two components of the quasispecies framework suggest that a permanently elevated mutation rate may be deleterious to the virus even when extinction is not achieved. First, a higher mutation rate reduces mean fitness around any local fitness peak. Second, a high mutation rate “flattens” the fitness landscape, so that some narrow peaks cannot be attained. Thus, a sustained high mutation rate that does not drive the virus to extinction may enforce a low mean fitness. Holmes [33] has likewise argued that the high mutation rates of RNA viruses may constrain adaptation. Without greater insight into the structure of biological fitness landscapes, we cannot yet assess whether high mutation rates will expedite or thwart adaptation. Evolutionary theory suggests that either outcome is possible.

In light of this potential impact of high mutation rates, it seems paradoxical that a new class of drugs is being touted for its ability to reduce mutation rates, and thereby block the evolution of resistance to other drugs [34]. Yet mutation reduction and lethal mutagenesis may both be feasible strategies for controlling a virus, with the two methods working at opposite ends of the evolutionary spectrum. Mutation suppression may thwart evolutionary exploration of the fitness landscape while mutation enhancement may overwhelm the genome with lethal mutations. There is necessarily a wide zone of mutation rates between these extremes that allow viral persistence.

## How to Demonstrate an Error Catastrophe

We have used a variety of simplifications to explain and depict error catastrophes from a mathematical perspective. Yet it may not be obvious how an error catastrophe would manifest itself in a population, especially how it would appear in a sample of genome sequences taken before and after crossing the error threshold. One possible scenario is similar to that of Figure 10: the error catastrophe is the deterministic accumulation of mildly deleterious nucleotides in a genome while preserving sites that are functionally essential. Figure 10 does not assign different fitness effects among loci but is easily extended to that case. The population will suffer the mutational loss of all nucleotides that cannot be maintained under the high mutation rate, while maintaining only those for which the fitness cost of a mutation is sufficiently large. This is similar to Kondrashov's version of Muller's ratchet, in which high fitness alleles ratchet out of the population until either the remaining alleles are sufficiently robust to mutation or all viable alleles have disappeared [35]. The different neutral networks in this process are neighbors in sequence space and differ by one or a few deleterious mutations. Thus the error catastrophe is observed as a population-wide change in nucleotide frequencies at possibly very few sites in the genome.

A second scenario is that of a population in which each individual's genome belongs to either of two “disjoint” neutral networks from very different parts of the fitness landscape. This type of polymorphism could arise if one type was present in the starting population and the other was introduced from a different population through migration. For this scenario, we assume that the population size is sufficiently small relative to the mutational distance between the two neutral networks, so that it is essentially impossible for the population to reach one network from the other via mutation (as modeled in some of the “survival of the flattest” work cited above). If one of the networks is more mutationally robust but of lower fitness than the other, an error catastrophe can cause the loss of the higher-fitness network. This type of error catastrophe might reveal itself in genomic data as the loss of polymorphism through the independent mutational meltdown and subsequent disappearance of one of the original types. Back mutation complicates the previous scenario by continually re-introducing the fitter nucleotides, which are then maintained at only a slightly higher level than neutral nucleotides. Back mutation is not a factor in the disjoint network model because the first network to disappear is exceedingly unlikely to be re-introduced via point mutations from the remaining population.

Given the difficulty of demonstrating the relatively simple properties of quasispecies dynamics and equilibrium properties, it is not surprising that the empirical demonstration of an error threshold/catastrophe has not been achieved. An interesting step in this direction was provided recently in a study that estimated the error threshold mutation rate that might apply to a primitive organism consisting of RNA molecules—a riboorganism [36]. The study concluded that an error catastrophe might be avoided in a substantially larger genome than had previously been thought. This result stemmed in part from the high degree of neutrality inferred from existing functional RNA molecules.

The systems offering the best hope for empirically demonstrating an error threshold are in fact naked RNA molecules evolved by directed evolution: ribozymes (catalytic RNA molecules) and aptamers (binding molecules) evolved in vitro from initial pools of random-sequence molecules. In this work, mutational robustness is recognized as a key property of molecules affecting evolution (e.g., [37]), but the concept has been approached from the perspective of adaptive evolution rather than error catastrophes (the Kun et al. study [36] cited above is unusual in applying the perspective of error catastrophes to ribozymes). A sequence “motif” is the set of bases at specific positions in the RNA molecule essential for function; changes in those bases destroy function but changes at other positions have no major effect. When there exist both short and long sequence motifs that are functionally similar, then, by simple combinatorics, the short motif will attain higher representation in a random sequence library than the long motif will. Thus, short motifs are more likely to be selected at the outset, creating a bias known as the “tyranny of short motifs” [38]. Furthermore, since the length of a motif determines the number of potential mutational targets, short motifs will typically be more mutationally robust than long motifs. In subsequent rounds of mutagenesis and selection, this may create additional bias toward short motifs.

Suppose a long motif has slightly greater functionality (our fitness, *w_i_*) than a short motif. By virtue of differences in mutational robustness among the different motifs, there should be an error threshold mutation rate, above which the long motifs will be displaced by the short motifs. If one knows the fitnesses *(w_i_)* and the number of critical bases in the long and short motifs *(m_i_),* it should be possible to estimate the value of the error threshold by using 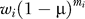 as the replacement rate of motif *i*. The ligase ribozymes seem like an appropriate model for such a calculation, because the class I ligases have a much longer motif (~70 bases) than the class II and class III ligases (~40 bases; [39]). Kinetic parameters of classes II and III were not determined for optimized ligase molecules, so it is not yet possible to estimate realistic fitness differences between the best members of each family, and even if kinetic properties were known, extrapolation to fitness is not trivial. Nonetheless, if all point mutations of critical bases are lethal and others are neutral, the error threshold would be a per base mutation rate of roughly 0.003 if class I had a fitness advantage of 10% over classII/III, a mutation rate of 0.01 for a 50% advantage, and a mutation rate of 0.06 for a 10-fold advantage of class I. An experimental demonstration of an error threshold may thus be feasible in this system.

The ligase ribozymes will permit an easier demonstration of an error threshold than most other empirical systems, because the alternative functional networks have been identified and can be seeded into the experiment. We thus know in advance what to compare. If, instead, we have information about only the wild-type but not the suboptimal type that dominates beyond the error threshold (as would be the case when working with viruses or bacteria), then it becomes far more complicated to identify an error threshold empirically. Even with the ligase ribozymes, if a population was initiated with only the class I molecule, the class II or III molecules might not evolve at high mutation rates because their sequences might be too distant in sequence space to arise in a quasispecies of class I molecules. All three classes arose in the initial experiment because they were selected from a random sequence pool rather than evolved from a single type.

Identification of an error threshold is certainly facilitated by use of a previously characterized genotype–fitness map, but that kind of information is not usually available. In ignorance of the alternative neutral networks for a phenotype, the obvious protocol to use in blindly testing for an error catastrophe is to impose stabilizing selection for the wild-type trait while progressively increasing the mutation rate. One would then test for characteristics of an error catastrophe, such as a population shift in genotype space or increased mutational robustness of the genotypes evolved at high mutation rate. The key difficulty in demonstrating an error catastrophe without a priori knowledge of alternative networks is that there are population genetic processes not considered in the simple quasispecies models that give rise to superficially similar outcomes. Thus, under our suggested experimental protocol, shifts in genotype space may stem from (i) an error catastrophe—the deterministic fixation of deleterious mutations, (ii) stochastic fixation of deleterious and neutral mutations, (iii) the higher mutation rate allowing the population to find higher adaptive peaks, or (iv) adaptive evolution because mutagenesis has altered the selective environment (other than through higher mutation rate). Likewise, increases in mutational robustness may, in fact, suggest an error catastrophe, or may simply be a by-product of a general decline in fitness under high mutation rates. Recent work suggests that genotypes of reduced fitness generally experience a higher fraction of mutations with beneficial effect than genotypes of high fitness [40,41]. This pattern may stem from the local structure of the fitness landscape: a genotype at a local optimum can experience no beneficial mutations, and the further a genotype lies from a peak, the greater the chance that a random mutation improves fitness. Even before sorting out these various interpretations, there are fundamental challenges to demonstrating that a shift in genotype space or in mutational robustness has actually occurred (e.g., Figure 10 shows some of the difficulties).

The theory of quasispecies error thresholds appears sound. The major avenues for new developments are twofold: (i) obtaining empirical support and (ii) incorporating additional realism into the basic models. There are several papers that make progress on this second point, by including recombination [17], stochastic effects [14], and the shape of the mutation–fitness function [15]. We still have much to learn about the relevance of error catastrophes to the natural world, and the effort promises to yield many new insights. 
